# Prevalence and Risk Factors of Surgical Site Infections in a Teaching Medical College in the Trichy District of India

**DOI:** 10.7759/cureus.39465

**Published:** 2023-05-25

**Authors:** Nivitha Mohan, Dhanalakshmi Gnanasekar, Sowmya TK, Anand Ignatious

**Affiliations:** 1 Microbiology, Aakash Multispeciality Hospital, Chennai, IND; 2 Microbiology, Dhanalakshmi Srinivasan Medical College and Research Centre, Chennai, IND; 3 Surgery, Trichy SRM (Sri Ramaswamy Memorial) Medical College and Research Centre, Trichy, IND

**Keywords:** hospital-based study, risk factors, microorganisms, prevalence, surgical site infection

## Abstract

Introduction

Surgical site infection (SSI) remains a common and widespread problem, which contributes to significant morbidity and mortality, prolongs hospital stays, and consequently increases healthcare costs. The current study aimed to assess the prevalence of SSI and its associated risk factors among patients who underwent any surgical intervention in a tertiary care center in Trichy, Tamil Nadu, India.

Methodology

This was a hospital-based, cross-sectional study that was carried out over a period of one year in Trichy, Tamil Nadu, India. All adult patients of both genders older than 16 years who underwent surgery were included. Patients who underwent second surgery at the same site for any reason, patients on immunosuppressant therapy or immunodeficiency disease, patients on antibiotics already, and patients with infection elsewhere were excluded. After 48 hours of surgery, if there was evidence of wound infection, then the patient was considered to have SSI. The data obtained were analyzed using SPSS (Statistical Package for the Social Sciences) version 21 (IBM Corp., Armonk, NY).

Results

A total of 2076 patients underwent different types of surgeries. The prevalence of SSIs during the study period was 5.6% (n = 2076). SSIs were more common in abdominal surgeries (61.2%). Patients aged 16-24 years have a higher risk of getting SSI than other age groups (p = 0.040). Males have a higher risk of getting SSI than females (p = 0.022). Patients who underwent emergency surgery have a higher risk of getting SSI than those who underwent elective surgery (p = 0.025). Those with diabetes had a higher risk of getting SSI than those who were non-diabetics (p ≤ 0.0001).

Conclusion

SSIs were more common in abdominal surgeries. Patients who are male, younger in age, had emergency surgery, have diabetes, and have had a long hospital stay are at a higher risk of developing SSIs after any kind of surgery.

## Introduction

Wound infection can be defined as the invasion of organisms through tissues following a breakdown of local and systemic host defenses, leading to cellulitis, lymphangitis, abscess, and bacteremia. Infections of surgical wounds are called surgical site infections (SSIs) [1]. SSIs are defined as infections occurring within 30 days after surgery or within one year if an implant is left in place after the procedure and affects either the incision or deep tissue at the operation site [2]. According to the National Nosocomial Infection Surveillance Program (NNIS), it is classified into superficial, deep, and organ/space infections [3].

Sources of SSIs include the patient’s own normal flora, organisms present in the hospital environment that are introduced into the patient by medical procedures, specific underlying diseases, trauma, or burns that may cause a mucosal or skin surface interruption [4]. SSIs are serious operative complications that occur in approximately 2% of surgical procedures and account for 20% of healthcare-associated infections. Many studies have reported that SSIs rank third among common nosocomial infections, next to the urinary tract and respiratory tract infections [2,5]. Recent studies reported that the SSI rate ranges from 19.4% to 36.5% all over the world, whereas it ranges from 3% to 12% in India [6-8].

SSI remains a common and widespread problem, which contributes to significant morbidity and mortality, prolongs hospital stays, and consequently increases healthcare costs. Factors that promote SSIs include length of hospital stay, obesity, diabetes mellitus, smoking, etc. The development of a postoperative wound infection depends on the complex interplay of many factors. For most postoperative wounds, the source of infection is endogenous. Exogenous infections are mainly acquired from the nose or skin flora of the operating team and transmitted through the hands of the surgeon or improper operation theater sterilization, which includes preoperative, intraoperative, and postoperative care [9]. Some significant factors that can influence the incidence of subsequent infection are surgical techniques, skin preparation, timing, the method of wound closure, and antibiotic prophylaxis after certain types of surgery. Also, many other factors have been identified as having an effect on the potential for infection, and these should be considered by healthcare professionals before, during, and after surgery [10].

The modern surgeon cannot escape the responsibility of dealing with infections and, when dealing with them, should have knowledge of the appropriate use of aseptic and antiseptic techniques, the proper use of prophylactic and therapeutic antibiotics, and adequate monitoring and support with novel surgical and pharmacological modalities as well as nonpharmacological aids [11]. With the above background, the current study was conducted with the aim of assessing the prevalence of SSI and its associated risk factors among patients who underwent any kind of surgery (both elective and emergency) in a tertiary care center in Trichy, Tamil Nadu, India.

This original research article had been submitted as a dissertation titled "Bacteriological profile antibiogram and risk factors of SSIs in a tertiary care hospital" on October 15, 2018, to the Tamil Nadu Dr. M.G.R. Medical University, Chennai.

## Materials and methods

Study settings and duration

This was a hospital-based, cross-sectional study that was carried out at the Department of Microbiology at a tertiary health care center at Irungalur, Trichy, India. The study was carried out over a period of one year (from May 1, 2017, to April 30, 2018).

Study population and sampling technique

As per the convenience sampling technique, all the cases admitted to the surgical wards (including both elective and emergency surgery) during the study period and those who met the eligibility criteria were included in the study.

Sample size calculation

The prevalence of SSI observed in the study by Tabiri et al. was 11.5% [12]. Based on this study, considering p as 11.5% and 95% confidence interval with the precision (d) of 2%, the sample size was calculated using the following formula:

$n\ =\ \frac{3.84\ \times (PQ)}{d2}$, where Q = 1-P.

The minimum sample to be included in the study was 978 patients. The total sample collected in this study was 2076 patients.

Inclusion criteria

All patients of both genders above 16 years who underwent surgery and were admitted to the surgical wards during the study period were included in the present study.

Exclusion criteria

All pediatric cases were excluded from the study. Patients who underwent second surgery at the same site for any reason, patients on immunosuppressant therapy or any known immunodeficiency disease, patients on antibiotics already for any other infections, and patients with infection elsewhere in the body were also excluded from the study.

Ethical clearance

The study was carried out after getting ethical approval from the Institutional Research Board and Institutional Ethics Committee (approval number: CMCH&RC/IEC - No 28 - 11.04.2017). Informed and written consent was obtained from every study subject.

Data collection procedure

Data about the age of the patients, gender, demographic details, clinical details including the name of the procedure, date and duration of surgery, the experience of surgeons, preoperative hospital stay, nature of the surgery, postoperative hospital stay, and the onset of illness (SSI) were collected by reviewing the patient’s case sheet.

After 48 hours of surgery, the dressings on the surgical wounds were removed. Evidence of wound infection was considered if the patient had local inflammatory changes such as edema, redness, warmth, or discharge from the wound site. The SSI was diagnosed and confirmed clinically by a registered medical practitioner. If there was any discharge, samples were collected before dressing the wounds. If only inflammatory changes were present without any discharge, the wounds were monitored for the development of discharge from the wound until the patient was sent home. If inflammatory signs were noticed within 48 hours, the patients were followed up with the help of their respective surgeons. In addition, these patients were educated and followed up via mobile phone for the development of SSIs over a period of 30 days.

The suspected wound infections were cleaned with sterile normal saline, followed by 70% alcohol, and then the specimen was collected using a sterile swab. Two swabs were taken from the depth of the wound, and/or the aspirates were collected in a sterile disposable syringe and transported to the laboratory within two hours. The color, consistency, and odor of the samples were observed and recorded.

A direct thin smear was made from each wound swab and/or aspirates on a clean grease-free glass slide and was air dried. It was then heat-fixed, and Gram staining was done with positive and negative control (American Type Culture Collection [ATCC] *Staphylococcus aureus* 25923 and *Escherichia coli* 25922). The presence of pus cells and microorganisms was observed under the oil immersion (100X) objective. The samples were cultured onto nutrient agar, 5% sheep blood agar, and MacConkey agar plates by adopting standard microbiological techniques. After 24 hours of incubation aerobically at 37°C, plates were read, and the isolates were identified based on colony morphology, Gram stain, motility, and biochemical tests.

Data analysis

The data obtained were entered in Microsoft Excel (Microsoft Corp., Redmond, WA), and the results were analyzed using SPSS (Statistical Package for the Social Sciences) version 21 (IBM Corp., Armonk, NY). All the data collected in the current study was categorical, so they were expressed in a table as frequency and percentage. Also, the figures were expressed as a pie chart. The association between risk factors and the presence of SSI was assessed using the Chi-square test. With a 95% confidence interval, a p-value of less than 0.05 was considered statistically significant.

## Results

A total of 2076 patients underwent different types of surgeries, including elective as well as emergency procedures, during the study period. About 116 SSIs were documented, and hence, the overall prevalence of SSI rate during the study period was 5.6% (n = 2076).

The number of cases that developed SSIs in relation to the type of surgery is shown in Table 1. Among the 2076 surgeries, abdominal surgeries constituted 35.6%. The highest prevalence rate of SSI occurred in the category of exploratory laparotomy (about 78 patients underwent exploratory laparotomy, and about 20 patients developed SSIs, which was about 25.6%).

**Table 1:** Prevalence of SSI according to the types of surgery (n = 2076) SSI: Surgical site infections.

Site of Surgery	Types of Surgeries	No. of Surgeries, n (%)	SSI, n (%)
Abdomen	Appendectomy	82 (3.94%)	13 (15.6%)
	Hernia repair	86 (4.14%)	16 (18.6%)
	Exploratory laparotomy	78 (3.7%)	20 (18.6%)
	Cholecystectomy	67 (3.22%)	12 (17.9%)
	Lower segment cesarian section	266 (12.8%)	6 (2.2%)
	Hysterectomy	160 (7.7%)	4 (2.5%)
Pelvis	Sphincterotomy	43 (2.0%)	2 (4.6%)
	Hemorrhoidectomy	41 (1.97%)	4 (9.7%)
	Fistulectomy	39 (1.87%)	2 (5.1%)
	Hip replacement	31 (1.58%)	6 (19.3%)
Urogenital	Transurethral resection of prostate	25 (1.2%)	2 (8%)
	Urethroscopy lithotripsy	66 (3.17%)	Nil
Breast and axilla	Modified radical mastectomy	24 (1.1%)	3 (12.5%)
	Fibroadenoma excision	61 (3.02%)	Nil
Skin, bone, and joints	Knee replacement	47 (2.26%)	4 (8.5%)
	Varicose vein	41 (1.97%)	Nil
	Open reduction and internal fixation	214 (10.3%)	13 (6.0%)
Eye	Intraocular lens implantation	454 (21.8%)	Nil
Ear, nose, throat	Tonsillectomy	123 (5.92%)	2 (1.6%)
	Mastoidectomy	96 (4.62%)	Nil
Neurosurgery		32 (1.54%)	Nil
Total		2076	116

Table 2 describes the proportion of various organisms isolated from the various surgical sites. All the organisms were isolated in high proportion in abdominal surgeries, which ranged from 43% to 100%.* Enterococci spp*.,* Citrobacter spp*.,and *Enterobacter spp*.were isolated 100% from abdominal surgeries. *Pseudomonas aeruginosa and Acinetobacter baumannii *were the two organisms that were isolated from various surgical sites.

**Table 2:** Proportion of various microsomal organisms isolated from the various surgical site infections (n = 116)

Organisms (n)	Abdomen (%)	Orthopedics (%)	Pelvis (%)	Breast (%)	Ear, Nose, and Throat (%)	Urology (%)
*Staphylococcus aureus* (32)	43	15	19	9	12	0
*Enterococci species* (3)	100	0	0	0	0	0
*Escherichia coli* (27)	70	30	0	0	0	0
*Klebsiella species* (19)	53	26	21	0	0	0
*Proteus species* (7)	57	29	0	0	14	0
*Citrobacter species* (1)	100	0	0	0	0	
*Enterobacter species* (7)	100	0	0	0	0	0
*Pseudomonas aeruginosa *(19)	53	17	10	0	10	10
*Acinetobacter baumannii* (9)	44	22	22	0	12	0

Table 3 describes the risk factors of the study population according to the SSI. Among the study subjects, about 28% of those aged 55 or older were diagnosed with SSI (the proportion of patients aged more than 55 years was higher than other age groups), and the second most common age group was 35-44 years (25%). Most cases of SSI were diagnosed in males (72%). Most of the SSIs were diagnosed in emergency surgeries (81%). The SSI has been categorized into superficial, deep, and organ SSIs. In the present study, it was observed that 69 (59%) had superficial SSI, and the rest (n = 47) had deep ones. Those who have been classified as American Society of Anesthesiologists (ASA) 3 had a higher proportion of SSI (51%). Among the individuals with diabetes mellitus, about 55% of them developed SSI. Among those who had a habit of smoking or alcoholism, about 42% and 35% of them developed SSI, respectively. Similarly, of those who stayed more than seven days, 76.7% of them were diagnosed with SSI. Among 2076 patients, 1307 underwent clean surgeries, and of these, 42 developed SSI (3.2%). The occurrence of SSIs among clean contaminated (n = 519), contaminated (n = 187), and dirty wounds (n = 63) were 5.2%, 11.2%, and 41.2% respectively.

**Table 3:** Distribution of risk factors of the study population according to SSI (n = 116) SSI: Surgical site infections.

S. No	Risk Factors		Frequency of SSI	Percentage
1	Age group (years)	16-24	13	11
		25-34	16	14
		35-44	29	25
		45-54	25	22
		>55	33	28
2	Gender	Male	84	72
		Female	32	28
3	Type of surgery	Emergency	94	81
		Elective	22	19
4	Extend of wound	Superficial	69	59
		Deep	47	41
		Organ	0	0
5	ASA (American Society of Anesthesiologists)	I	16	14
		II	35	30
		III	59	51
		IV	6	5
		V	0	0
6	Diabetes mellitus	Yes	64	55
		No	52	45
7	Smoking	Yes	49	42
		No	67	58
8	Alcoholism	Yes	41	35
		No	75	65
9	Anemia	Yes	31	27
		No	85	73
10	Hospital stay	1-7 days	27	23
		>7 days	89	77
11	Drain	Yes	18	16
		No	98	84

**Figure 1:**
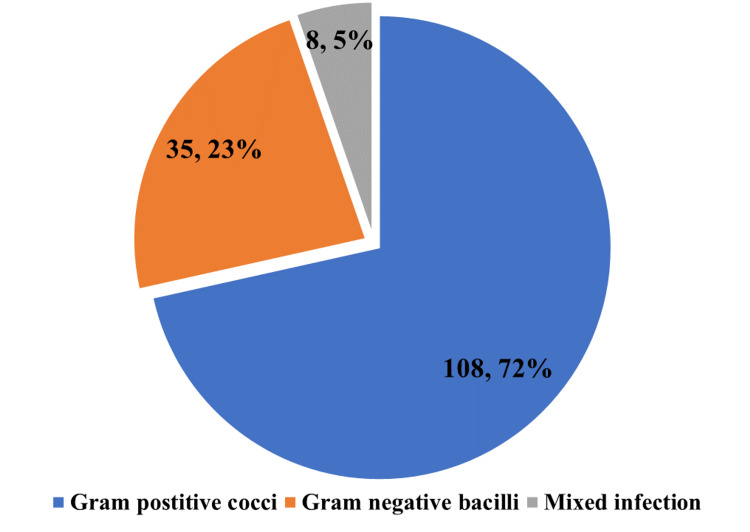
Distribution of organisms found in SSI according to the Gram staining (n = 124) SSI: Surgical site infections.

Figure 1 depicts the distribution of organisms found in SSI according to the Gram staining. A major proportion of the infection was caused by Gram-negative bacilli which was 72%.

The association between the risk factors and SSI infection among the study participants was described in Table 4. The lower age group of 16-24 years has a higher risk of getting SSI than other age groups according to the Chi-square test (p = 0.040). Males have a higher risk of getting SSI than females according to the Chi-square test (p = 0.022). Patients who underwent emergency surgery have a higher risk of getting SSI than those who underwent elective surgery according to the Chi-square test (p = 0.025). Patients who had diabetes had a higher risk of getting SSI than those who were non-diabetics according to the Chi-square test (p < 0.0001). Patients who had a smoking habit had no association with SSI (p = 0.526). Patients who had a habit of consuming alcohol had no association with SSI (p = 0.822). Patients who had a hospital stay longer than seven days have a higher risk of getting SSI than those who stayed less than or equal to seven days according to the Chi-square test (p < 0.0001).

**Table 4:** Association between the risk factors and SSIs among the study participants (n = 2076) SSI: Surgical site infections.

Risk Factors		SSI infections		Chi-Square Value	P-Value
		Yes	No		
Age group (years)	16-24	13 (12)	96 (82)	9.992	0.040
	25-34	16 (7)	221 (93)		
	35-44	29 (6)	527 (94)		
	45-54	25 (5)	476 (95)		
	>55	33 (5)	640 (95)		
Gender	Male	84 (7)	1213 (93)	5.176	0.022
	Female	32 (4)	747 (96)		
Type of surgery	Elective	94 (5)	1726 (95)	5.001	0.025
	Emergency	22 (10)	234 (90)		
Diabetes mellitus	Yes	64 (14)	386 (86)	80.737	<0.0001
	No	52 (3)	1568 (97)		
Smoking	Yes	49 (5)	887 (95)	0.401	0.526
	No	67 (6)	1073 (94)		
Alcohol	Yes	41 (5)	713 (95)	0.050	0.822
	No	75 (6)	1247 (94)		
Hospital stay	1-7 days	27 (2)	1612 (98)	229.15	<0.0001
	>7 days	89 (20)	348 (80)		

## Discussion

In our study, 2076 patients underwent various surgeries. Among them, 739 patients underwent various abdominal surgeries like exploratory laparotomies, hernia repairs, appendicectomies, hysterectomies, etc. A total of 302 patients had undergone orthopedic procedures like open reduction and internal fixation (ORIF), hip and knee replacement, etc., and 154 and 85 had undergone pelvic and breast surgeries, respectively. A study done by Allegranzi et al. [13] also reported that abdominal surgeries are commonly done and have high rates of SSIs. In contrast to our study, a study done by Maksimović et al. reported that orthopedic surgeries were more commonly associated with SSI [14]. Yet, this discussion needs further research.

About 134 patients showed local signs and symptoms and were suspected to have postoperative wound infections. These cases were evaluated and followed up. Among them, the culture was positive in 116 (5.5%) cases and hence was considered as cases of SSI in our hospital; thus, the overall prevalence rate of SSIs was 5.5%. In contrast, Kumar et al. [8] and Al-Mulhim et al. [15] reported in their study that the overall prevalence rate of SSIs was 2.5%, which is only half of our present study rate. The current status of SSIs identified in their hospital concurs with the studies of Golia et al. [16], and Iqbal et al. [17], who reported it as 4.3%, 5.4%, and 7.3%, respectively. On the contrary, Setty et al. [18] reported it as 21.66% and 22.2%.

The occurrence of SSIs in the present study was more in males (6.4%) when compared to females (4.1%), and it is statistically significant. A study by Hernandez et al. in 2005 conducted in a Peruvian Hospital reported more occurrences among males (65.6%) [19]. In contrast, a study done by Shanmugam et al. reported almost equal occurrences among females (52%) and males (48%) [20]. The increasing occurrence among males was attributable to the nature of the infected wounds with which they come to surgical departments. In the present study, the distribution of SSIs among the study participants aged 25 years and above was almost nearer to each other and varied from 4.9% to 6.7%. On the contrary, SSI was more among patients of age below 25 years. This might be attributable to the nature of the wound they acquired. But in general, the occurrence of SSIs was more as age advances since these cases were suffering from diabetes mellitus and/or other co-morbid conditions, which contribute to decreased physiological defense mechanisms and poor immune function. It is supported by many studies, for example, Owens et al. [21] and Bharatnur et al. [22] who reported that a greater number of SSIs occurred among 36-50 years (1.3 times higher risk of acquiring SSIs than the ones who were in the age group of 10-35 years). Similarly, a high rate of infection was noted in the later age groups by Mundhada et al. [23]. Our study findings challenge the above discussion and inferred the need for further research.

The present study includes 1820 elective surgeries and 256 emergency surgeries, and among them, 94 (5.6%) and 22 (8.59%) developed SSI, respectively. When the data was analyzed using a 2/2 table, it was noticed that the chances of development of SSIs were among emergency surgeries with a statistical significance (p = 0.025), and the odd’s ratio was found to be 0.57. The increased rate of SSI in emergency surgeries may be due to a very narrow time span without proper patient preparation and surgical preparedness as well as contaminated wounds as in cases of road traffic accidents. The same has been cited in most of the studies done earlier on SSIs. Tabiri et al. also reported that emergency cases had a higher number of SSIs (23.8%) as compared to elective cases (7.4%) [12]. In another study done by Dessie et al., SSIs were reported in 61.7% of emergency cases and 38.3% of elective cases [24].

In the present study, superficial and deep SSIs were 69 (59.4%) and 47 (40.5%), respectively. Superficial SSI was found to be higher. Kumar et al. [8] reported that superficial incision SSI was more prevalent (215 cases, 55.9%) followed by deep incisional SSI (169 cases, 44%), and van Walraven et al. [25] reported the same that a majority of these (n = 8188, 57.5% of all SSIs) had a superficial component. This is discordant with the study by Dessie et al., who reported superficial SSI as 42.1% and deep SSI as 57.9% (112 cases) [24].

In our study, 64 (55.1%) diabetic patients had SSI, which was higher than non-diabetics with statistical significance. The occurrence of SSIs among diabetics in the present study concurs with the study of Lilienfeld et al. [26] and Talbot [27], who reported that SSI among diabetics was 50%. In our study, 49 (42.2%) had a smoking habit, and 41 (35.3%) of 116 SSIs were alcoholics. The present observations were contradicted by Shabanzadeh and Sørensen [28] as well as Rantala et al. [29], who stated that alcohol did not affect SSIs.

Limitations of the study

This is a single-center study confined to aerobic bacterial pathogens only. In this study, pediatric cases were not included. Since this study was a cross-sectional study, the association found in this study did not need to be a causative factor. Certain factors such as secondary surgeries and preoperative preparation were not considered in this study.

## Conclusions

The prevalence of SSI among adult patients who underwent any kind of surgery in India was 5.6%. SSIs were more common in abdominal surgeries. Patients who are male, younger in age, underwent emergency surgery, have diabetes, and have a long hospital stay have a higher risk of developing SSIs after any kind of surgery. The most common organism encountered as SSI was *S. aureus* followed by *E. coli.*
